# Supplementary material for: Direct 1,3-butadiene biosynthesis in Escherichia coli via a tailored ferulic acid decarboxylase mutant
Source: Nat Commun. 2021 Apr 13;12:2195. doi: 10.1038/s41467-021-22504-6 (PMC8044207; doi:10.1038/s41467-021-22504-6)
Supplement: Supplementary file 1 — Supplementary Information [file 41467_2021_22504_MOESM1_ESM.pdf]

Direct 1,3-butadiene biosynthesis in *Escherichia coli via* a tailored ferulic acid decarboxylase mutant

Mori *et al.*

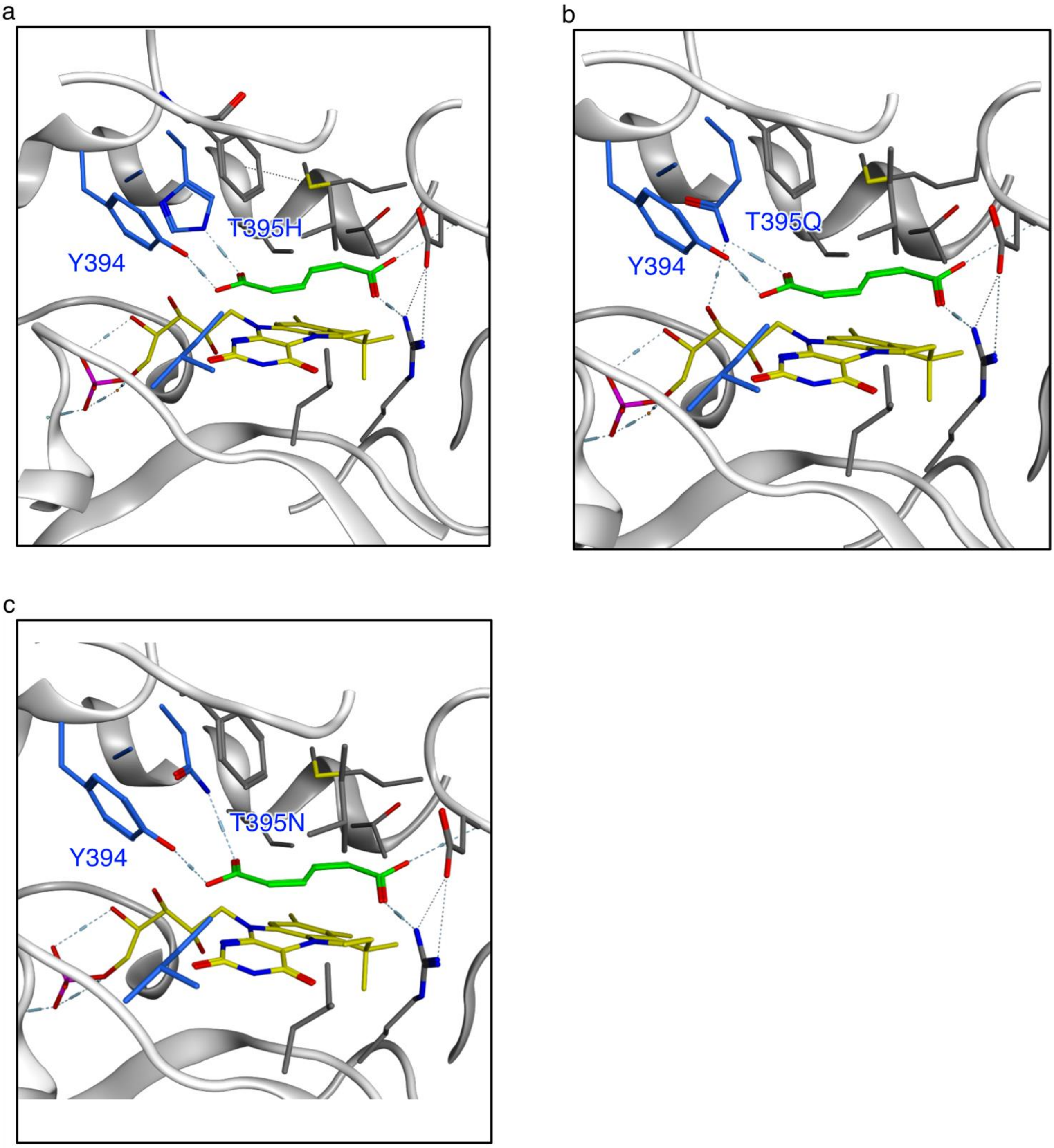

Supplementary Fig. 1. Simulated active site of *An*FDC mutants with bound *cc*MA. Designed **a** *An*FDC T395H, **b** *An*FDC T395Q, and **c** *An*FDC T395N. The negative hydrogen network and hydrophobic interactions are shown as dashed lines. *An*FDC, ferulic acid decarboxylase derived from *Aspergillus niger*; *cc*MA, *cis*,*cis*-muconate.

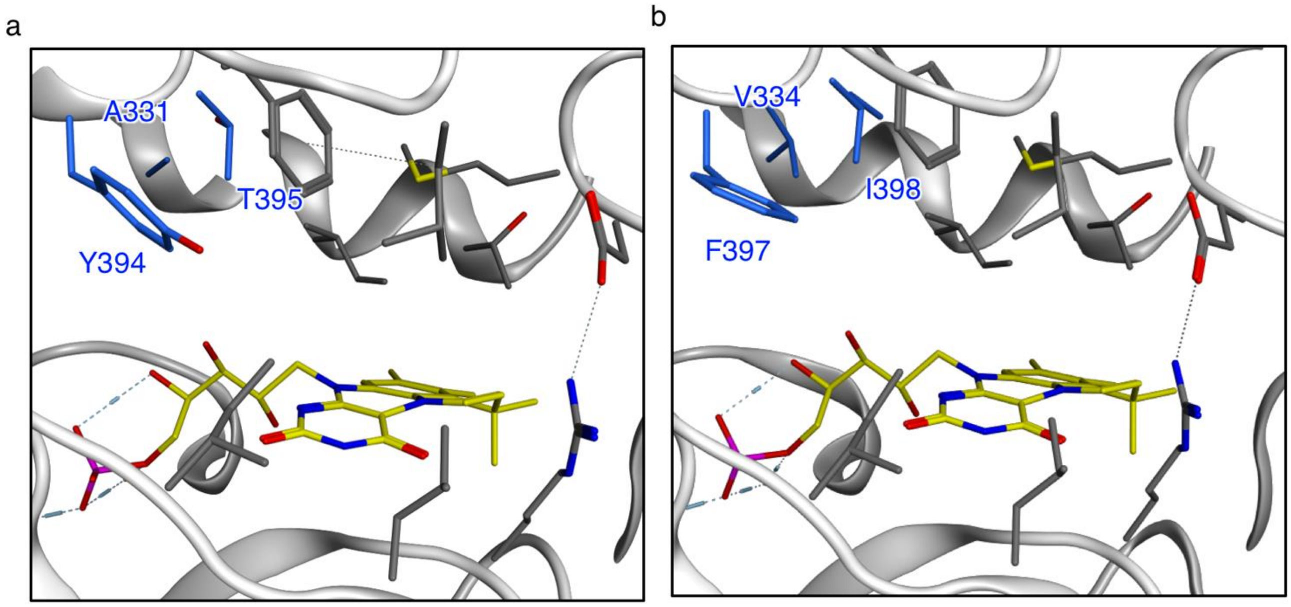

Supplementary Fig. 2. Active site of a *An*FDC and b *Sc*FDC. The negative hydrogen network and hydrophobic interactions are shown as dashed lines. *An*FDC, ferulic acid decarboxylase derived from *Aspergillus niger*; *Sc*FDC, ferulic acid decarboxylase derived from *Saccharomyces cerevisiae*.

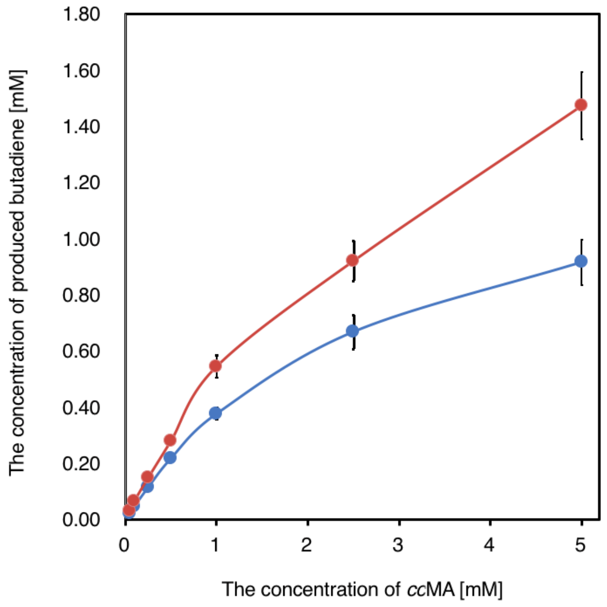

Supplementary Fig. 3. Comparison of decarboxylation activity between *An*FDC Y394H:T395Q and *Sc*FDC F397H:I398Q. Graph showing the amount of 1,3-butadiene produced by *An*FDC Y394H:T395Q (blue) and *Sc*FDC F397H:I398Q (red) at different *cc*MA concentrations after 24 h of culture. The data are presented as the means ± SDs of three independent experiments (n = 3). *cc*MA, *cis*,*cis*-muconic acid; SD, standard deviation. Source data are provided as a Source Data file.

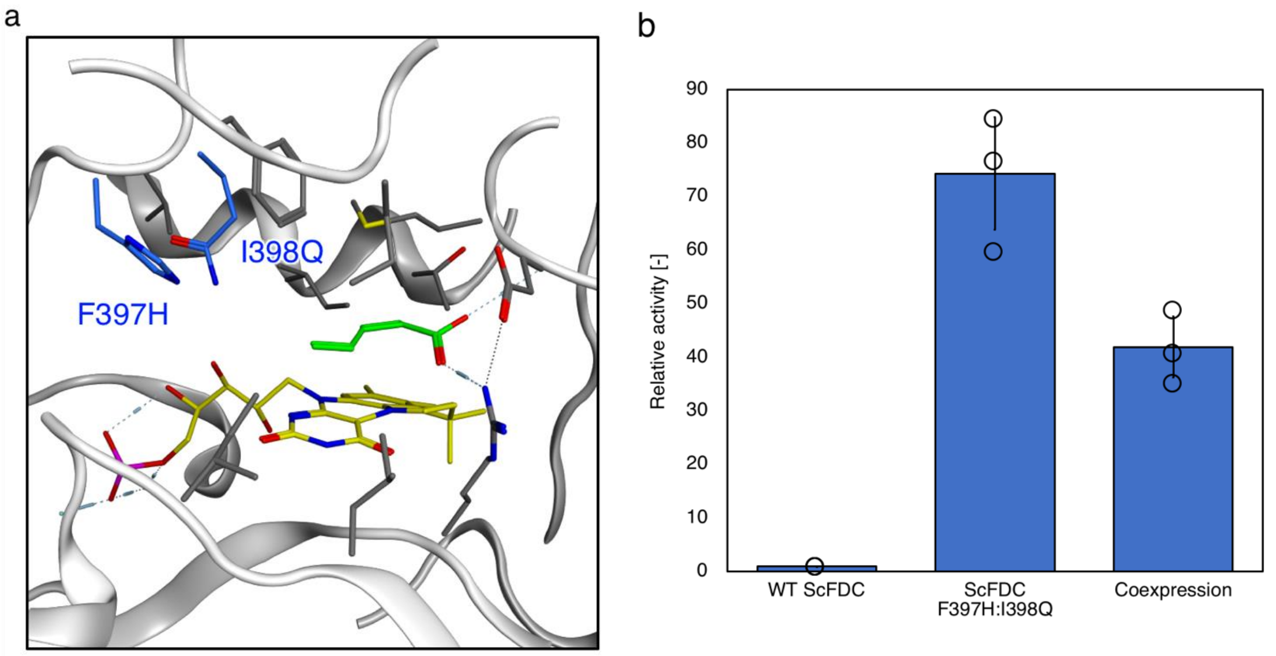

Supplementary Fig. 4. Active site of *Sc*FDC F387H:I398Q with bound PA (a) and decarboxylation activity of the combination of WT *Sc*FDC and *Sc*FDC F387H:I398Q (b). The negative hydrogen network and hydrophobic interactions are shown as dashed lines. The data are presented as the means ± SDs of three independent experiments (*n* = 3). PA, pentadienoic acid; WT, wild type; *Sc*FDC, ferulic acid decarboxylase derived from *Saccharomyces cerevisiae*; SD, standard deviation. Source data underlying Supplementary Figure 4b are provided as a Source Data file.

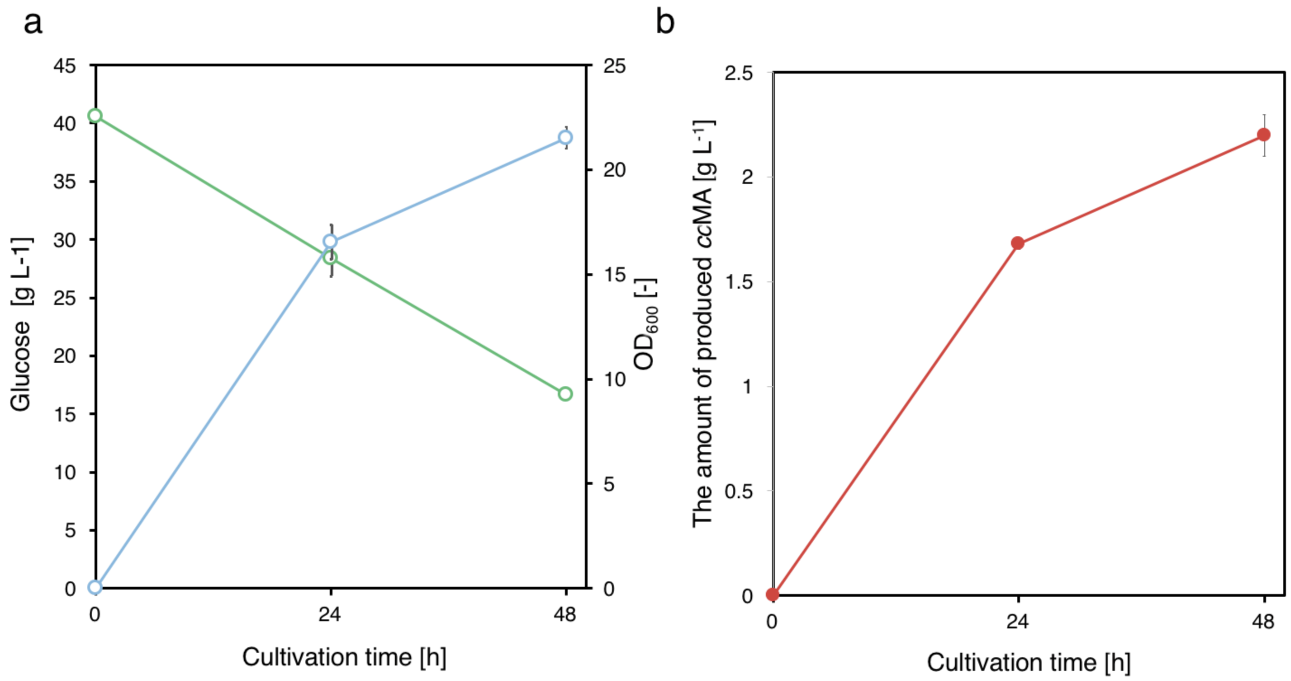

Supplementary Fig. 5. Culture profiles of the *cc*MA-producing CFB22 strain. Time courses of **a** bacterial cell growth (open blue circles) and glucose consumption (green open circles) and **b** the amount of *cc*MA produced (solid red circles). The data are presented as the means ± SDs of three independent experiments (n = 3). *cc*MA, *cis*,*cis*-muconic acid; SD, standard deviation. Source data are provided as a Source Data file.

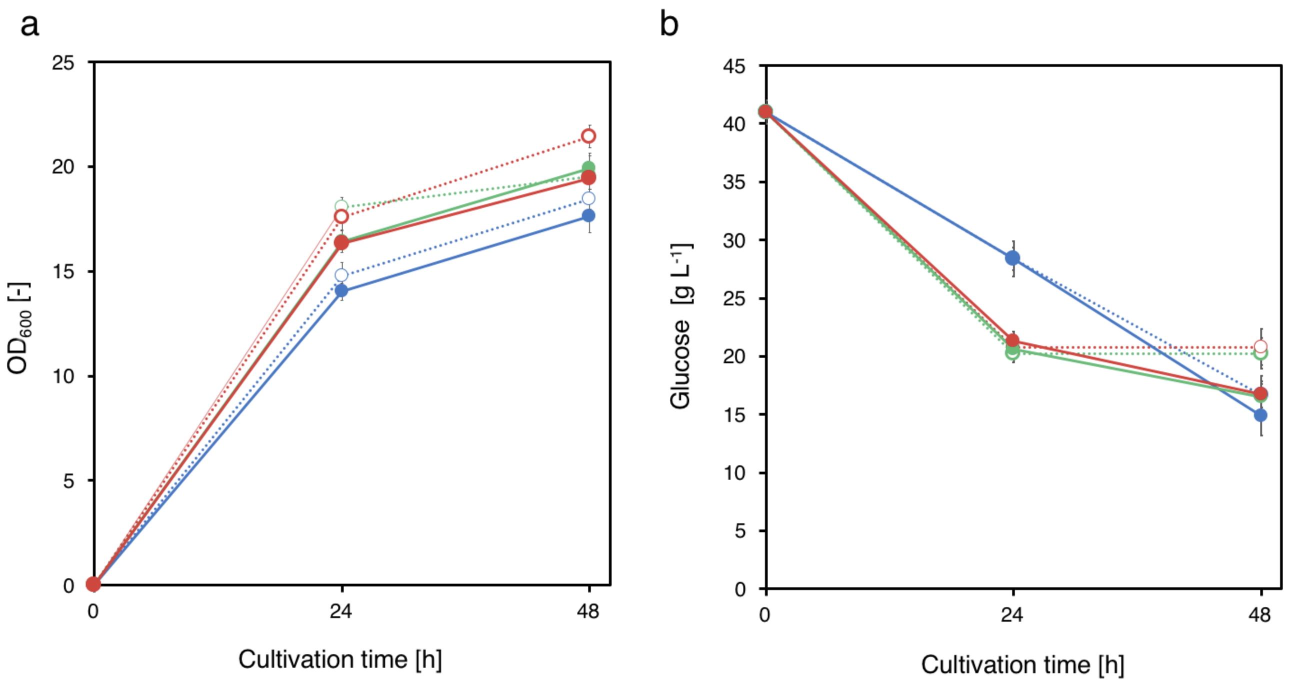

Supplementary Fig. 6. Culture profiles of the 1,3-butadiene-producing strains CFB210–CFB222 under aerobic conditions (for 24 h) and packed culture conditions (for another 24 h). Time courses of **a** bacterial cell growth and **b** glucose consumption. CFB210, open blue circles, dashed lines; CFB211, open green triangles, dashed lines; CFB212, open red squares, dashed lines; CFB220, solid blue circles, solid lines; CFB221, solid green triangles, solid lines; CFB222, solid red squares, solid lines. The data are presented as the means ± SDs of three independent experiments (*n* = 3). SD, standard deviation. Source data are provided as a Source Data file.

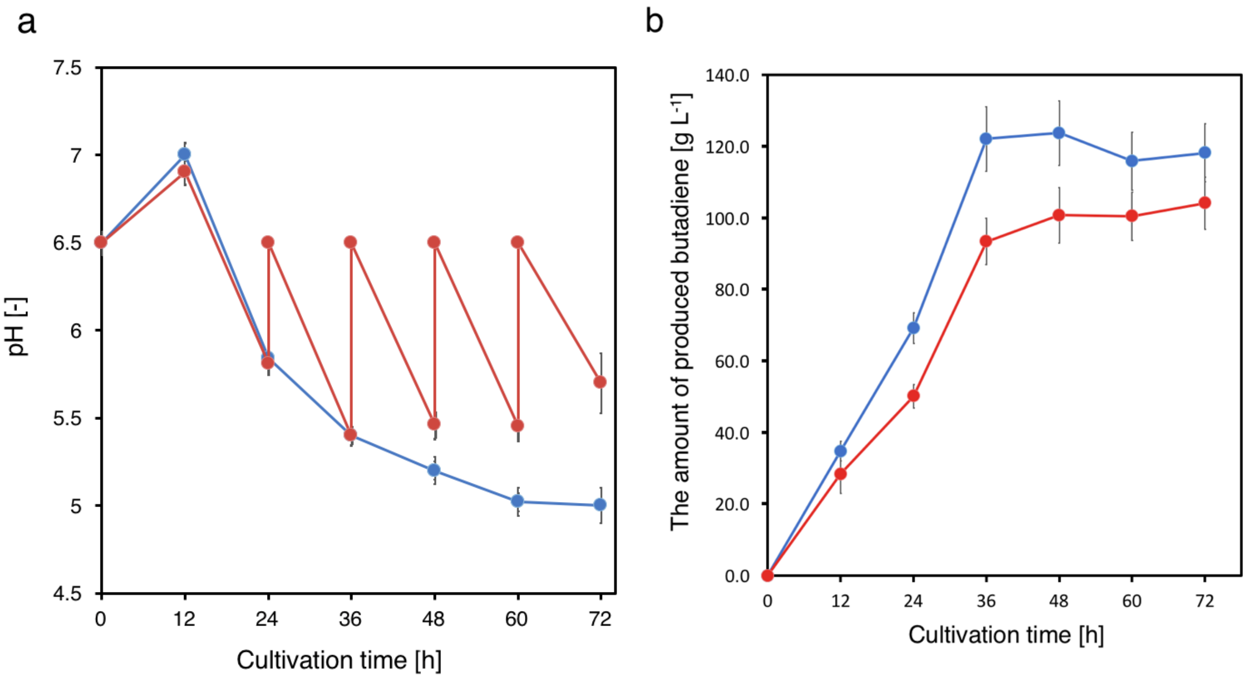

Supplementary Fig. 7. Culture profiles of the 1,3-butadiene-producing CFB222 strain under microaerobic condition. a Time courses of the pH and **b** 1,3-butadiene yield with (red) or without (blue) the pH adjusted to 6.5. The data are presented as the means ± SDs of three independent experiments (*n* = 3). SD, standard deviation. Source data are provided as a Source Data file.

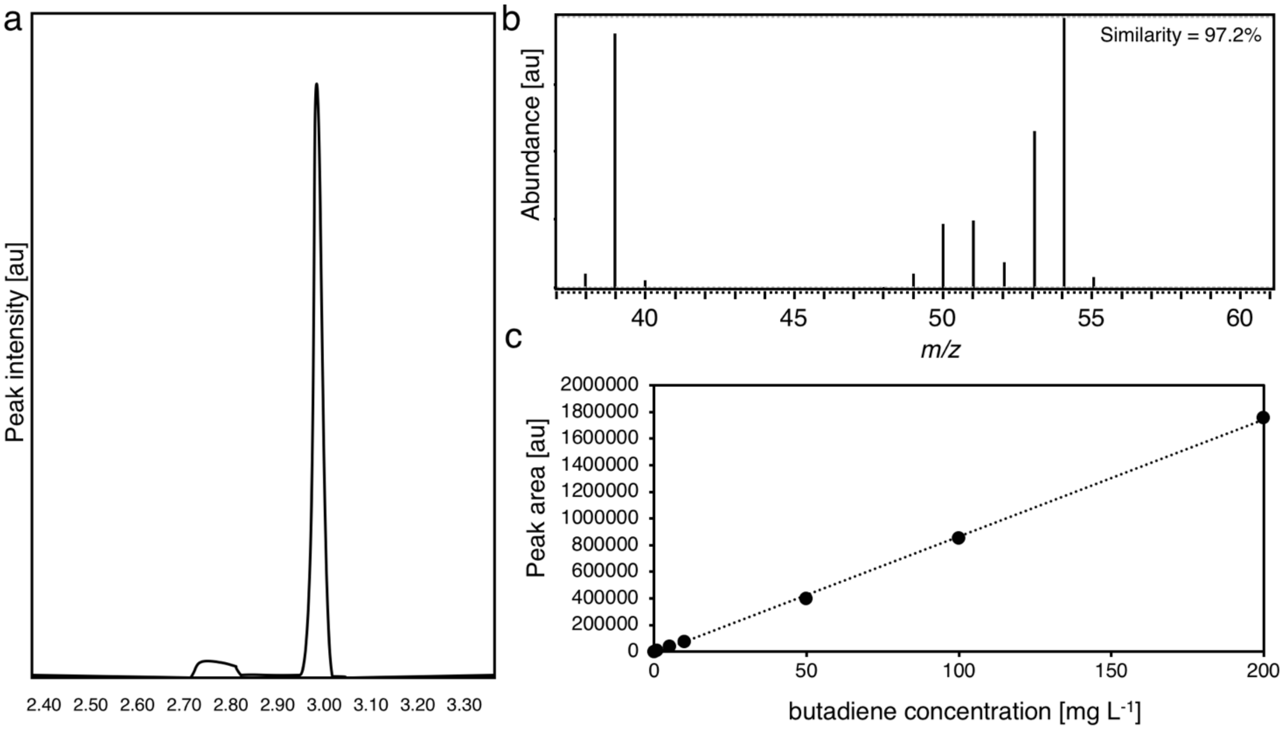

Supplementary Fig. 8. 1,3-butadiene analysis profiles. a Chromatogram and **b** mass spectrum via HS/GC–MS. **c** Calibration curve for 1,3-butadiene. HS/GC–MS, headspace/gas chromatography–mass spectrometry. Source data are provided as a Source Data file.

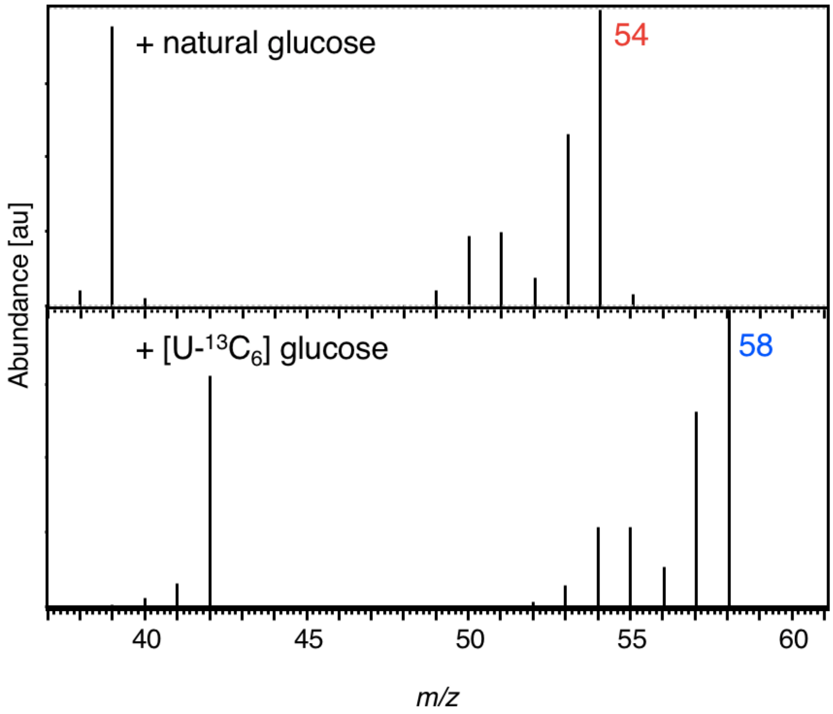

The relative area of each main peak is defined as 100 %

m/z	[M+0]	[M+1]	[M+2]	[M+3]	[M+4]
54	100.0	66.8	11.6	25.5	29.0
55	0.0	100.0	66.8	11.6	25.5
56	0.0	0.0	100.0	66.8	11.6
57	0.0	0.0	0.0	100.0	66.8
58	0.0	0.0	0.0	0.0	100.0

Supplementary Fig. 9. Mass spectrum of 1,3-butadiene produced during CFB222 culture when [U-$^{13}C_6$] glucose was used. Each peak area distribution was created on the basis of its 1,3-butadiene standard.

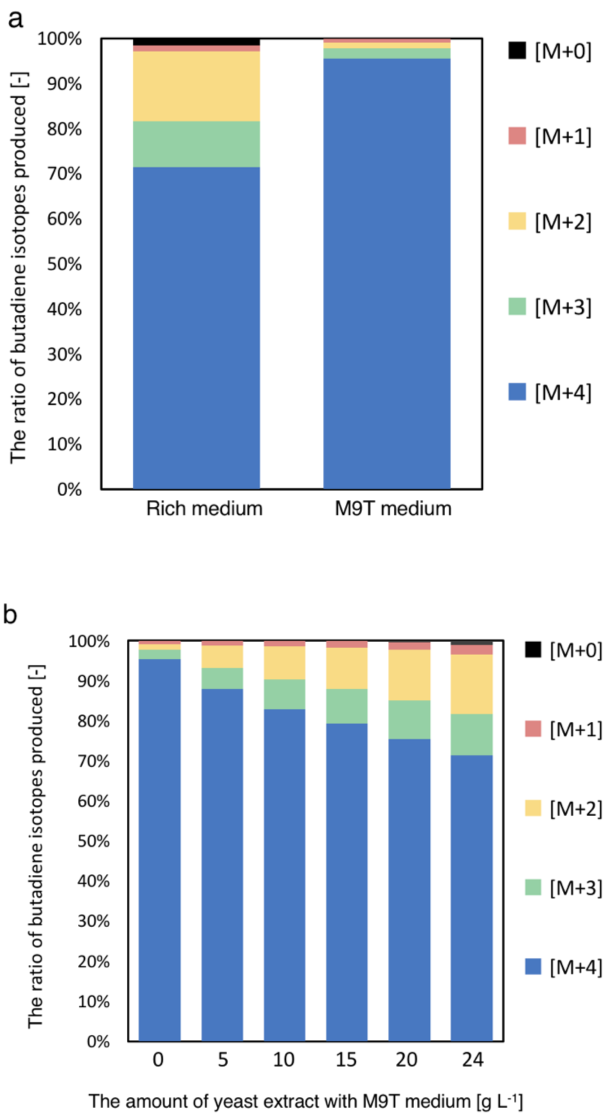

Supplementary Fig. 10. 1,3-Butadiene isotope profiles of CFB222 culture in different media when [U-$^{13}C_6$] glucose is used. **a** Ratio of butadiene isotopes produced when [U-$^{13}C_6$] glucose was used in rich media and M9T media. **b**, Ratio of butadiene isotopes produced when [U-$^{13}C_6$] glucose was used in M9T media with different concentrations of yeast extract. Source data are provided as a Source Data file.

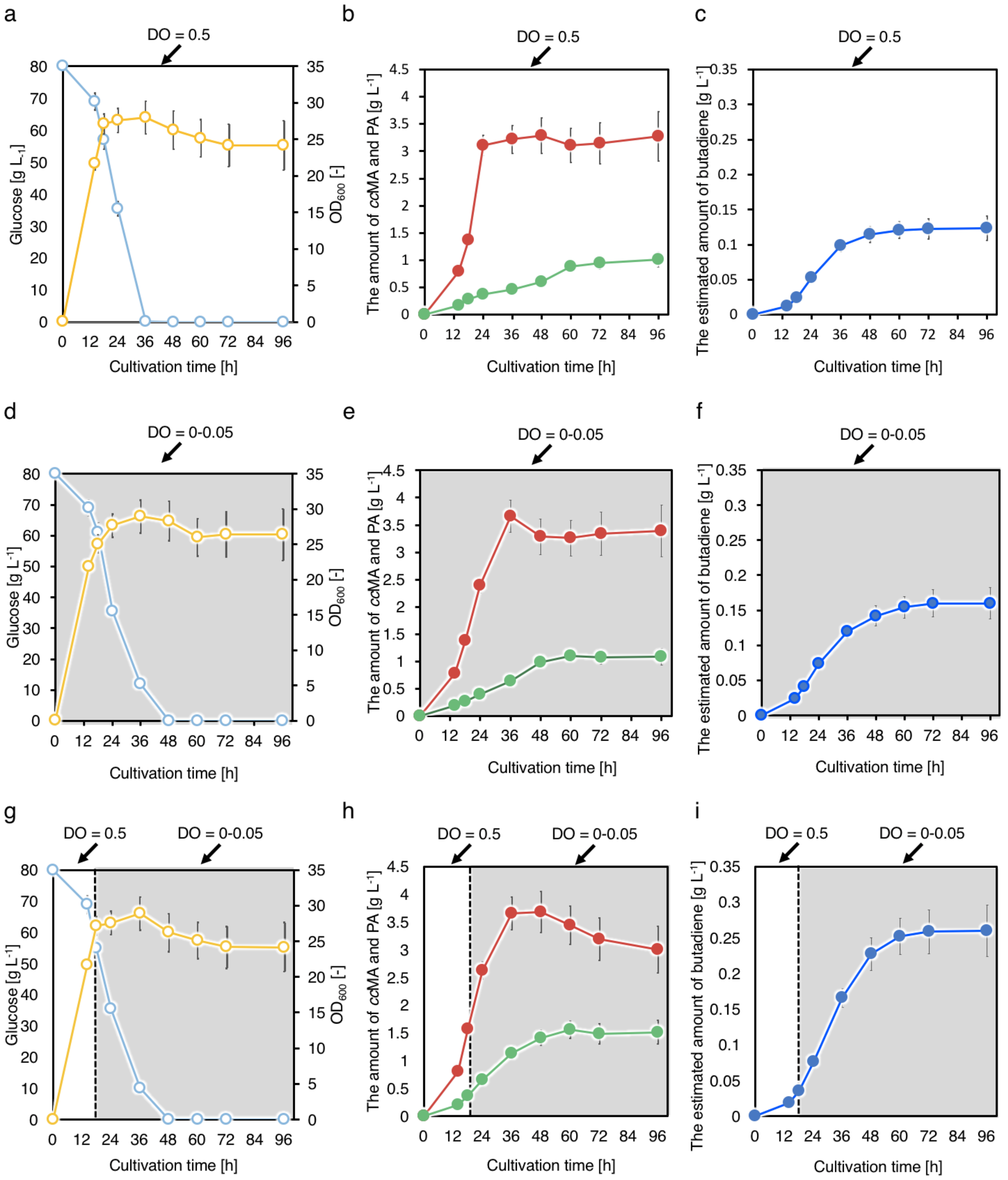

Supplementary Fig. 11. Batch culturing of CFB222 in a 1-L jar fermenter under three different DO conditions at a pH of 7.0. a, b, and **c** show the batch culture profile with DO maintained at 0.5 ppm (aerobic conditions) during the entire culture. **d, e,** and **f** show the batch culture profile with DO maintained at 0–0.05 ppm (microaerobic conditions) during the entire culture. **g, h,** and **i** show the batch culture with the DO shifted from 0.5 to 0–0.05 ppm at 18 h. **a, d, g** Time courses of bacterial cell growth (open yellow circles) and glucose concentration (open blue circles). **b, e, h** Time courses of *cc*MA (solid red squares) and PA (solid green triangles) yields. **c, f, i** Time courses of the estimated 1,3-butadiene yield (solid blue circles). The data are presented as the means ± SDs of three independent experiments (*n* = 3). *cc*MA, *cis*,*cis*-muconic acid; PA, pentadienoic acid; SD, standard deviation. Source data are provided as a Source Data file.

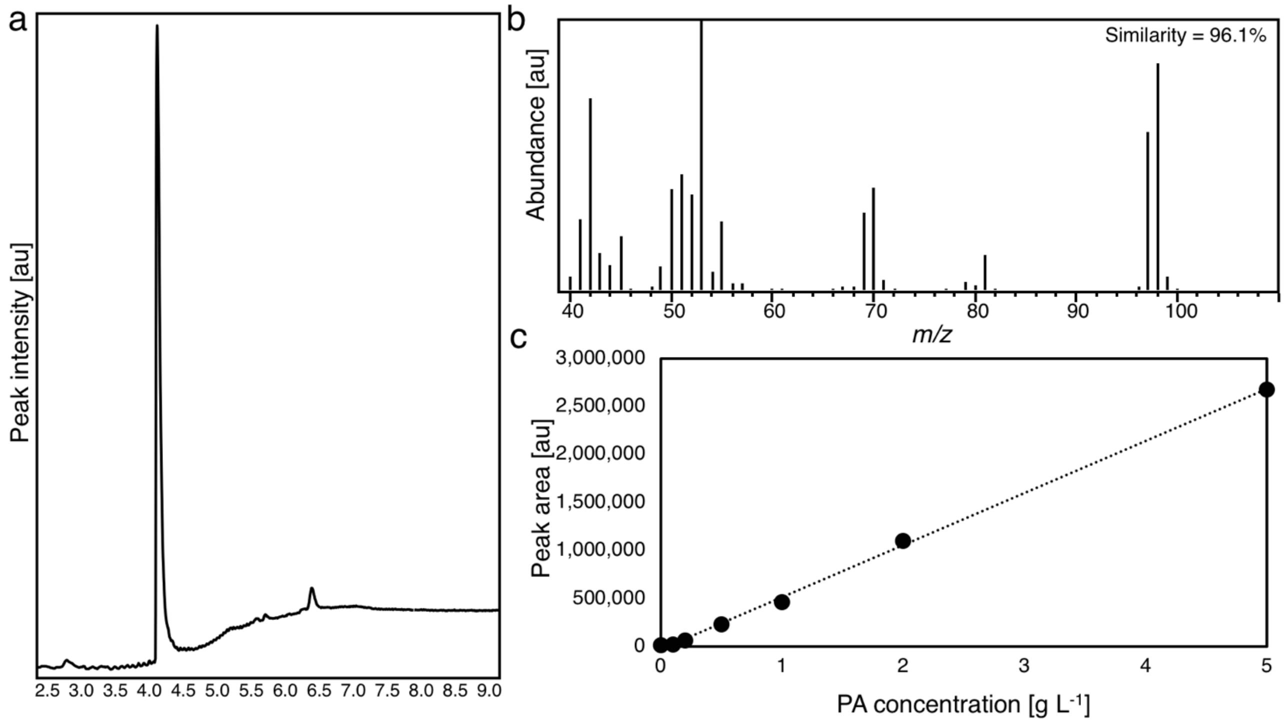

Supplementary Fig. 12. PA analysis profiles. a Chromatogram and **b** mass spectrum via GC-MS. **c** Calibration curve for PA. PA, pentadienoic acid; GC–MS, gas chromatography–mass spectrometry. Source data are provided as a Source Data file.

Supplementary Table 1. Sequences of the combinatorial library of multiple mutants.

Enzyme	Sequence			Enzyme	Sequence		
Wild type	I187	A331	Y394	AXX15	I187N	331T	Y394N
T395X_01	I187N	A331	Y394	T395X_16	I187N	331T	Y394Q
T395X_02	I187H	A331	Y394	T395X_17	I187H	331T	Y394
T395X_03	I187	A331T	Y394	T395X_18	I187H	A331T	Y394R
T395X_04	I187	A331	Y394R	T395X_19	I187H	A331T	Y394H
T395X_05	I187	A331	Y394H	T395X_20	I187H	A331T	Y394N
T395X_06	I187	A331	Y394N	T395X_21	I187H	A331T	Y394Q
T395X_07	I187	A331	Y394Q	T395X_22	I187H	331T	Y394R
T395X_08	I187N	A331T	Y394	T395X_23	I187H	331T	Y394H
T395X_09	I187N	A331	Y394R	T395X_24	I187H	331T	Y394N
T395X_10	I187N	A331	Y394H	T395X_25	I187H	331T	Y394Q
T395X_11	I187N	A331	Y394N	T395X_26	I187	331T	Y394R
T395X_12	I187N	A331	Y394Q	T395X_27	I187	331T	Y394H
T395X_13	I187N	A331T	Y394R	T395X_28	I187	331T	Y394N
T395X_14	I187N	A331T	Y394H	T395X_29	I187	331T	Y394Q

T395X: T395H, T395Q, or T395N was used as a template.

Supplementary Table 2. Relative activity of the combinatorial library of multiple mutants.

Enzyme	Relative activity	Enzyme	Relative activity	Enzyme	Relative activity
Wild type	1.0*				
T395H	67.5	T395Q	109.3	T395N	48.9
T395H_01	362.1	T395Q_01	142.1	T395N_01	57.2
T395H_02	131.1	T395Q_02	252.0	T395N_02	92.5
T395H_03	39.6	T395Q_03	119.3	T395N_03	39.5
T395H_04	6.5	T395Q_04	87.5	T395N_04	4.3
T395H_05	156.2	T395Q_05	1,002.1	T395N_05	135.7
T395H_06	77.7	T395Q_06	393.4	T395N_06	52.5
T395H_07	29.0	T395Q_07	206.9	T395N_07	39.6
T395H_08	335.8	T395Q_08	131.9	T395N_08	58.2
T395H_09	6.5	T395Q_09	22.2	T395N_09	4.9
T395H_10	476.5	T395Q_10	964.5	T395N_10	166.4
T395H_11	333.2	T395Q_11	347.7	T395N_11	65.6
T395H_12	79.5	T395Q_12	242.9	T395N_12	59.2
T395H_13	13.0	T395Q_13	10.9	T395N_13	10.0
T395H_14	472.9	T395Q_14	818.0	T395N_14	146.3
T395H_15	138.4	T395Q_15	370.2	T395N_15	68.7
T395H_16	0.0	T395Q_16	164.4	T395N_16	97.1
T395H_17	72.0	T395Q_17	346.5	T395N_17	141.1
T395H_18	6.6	T395Q_18	21.2	T395N_18	4.7
T395H_19	138.6	T395Q_19	990.4	T395N_19	58.2
T395H_20	119.0	T395Q_20	459.2	T395N_20	54.2
T395H_21	6.6	T395Q_21	195.8	T395N_21	65.6
T395H_22	13.4	T395Q_22	21.5	T395N_22	4.8
T395H_23	80.1	T395Q_23	841.5	T395N_23	97.7
T395H_24	72.2	T395Q_24	10.9	T395N_24	113.1
T395H_25	0.0	T395Q_25	130.1	T395N_25	98.6
T395H_26	6.4	T395Q_26	10.9	T395N_26	4.8
T395H_27	118.7	T395Q_27	972.7	T395N_27	104.2
T395H_28	53.6	T395Q_28	152.9	T395N_28	35.2
T395H_29	0.0	T395Q_29	129.4	T395N_29	75.0

WT, wild type; *An*FDC, ferulic acid decarboxylase derived from *Aspergillus niger*. * The activity of WT AnFDC was defined as 1.0. Source data are provided as a Source Data file.

Supplementary Table 3. Summary of the amount of glucose consumed and *cc*MA yield in CFB01, CFB11, and CFB21 cultures.

	Strains		
	**CFB01**	**CFB11**	**CFB21**
Consumed glucose (g L^{-1})	17.3	23.1	23.8
Produced *cc*MA (g L^{-1})	0.54	2.17	2.52
*cc*MA yield (mol mol^{-1})	0.040	0.119	0.134

*cc*MA: *cis*,*cis*-muconate. Source data are provided as a Source Data file.
