# Supplementary material for: Direct 1,3-butadiene biosynthesis in Escherichia coli via a tailored ferulic acid decarboxylase mutant
Source: Nat Commun. 2021 Apr 13;12:2195. doi: 10.1038/s41467-021-22504-6 (PMC8044207; doi:10.1038/s41467-021-22504-6)
Supplement: Supplementary file 7 — Description of Additional Supplementary Files [file 41467_2021_22504_MOESM7_ESM.docx]

**Description of Additional Supplementary Files**

File name: Supplementary Data 1
Description: List of strains and plasmids

File name: Supplementary Data 2
Description: List of primers

File name: Supplementary Data 3
Description: Sequence of FDC from *A. niger*.

File name: Supplementary Data 4
Description: Sequence of FDC from *S. cerevisiae*.
